# Editorial: Role of Nrf2 in Disease: Novel Molecular Mechanisms and Therapeutic Approaches

**DOI:** 10.3389/fphar.2019.01149

**Published:** 2019-10-04

**Authors:** Javier Egea, Águeda González-Rodríguez, Carmen Gómez-Guerrero, Juan Antonio Moreno

**Affiliations:** ^1^Molecular Neuroinflammation and Neuronal Plasticity Research Laboratory, Hospital Universitario Santa Cristina, Instituto de Investigación Sanitaria-Hospital Universitario de la Princesa, Madrid, Spain; ^2^Instituto Teófilo Hernando, Departamento de Farmacología y Terapéutica, Facultad de Medicina, UAM, Madrid, Spain; ^3^Centro de Investigación Biomédica en Red de Enfermedades Hepáticas y Digestivas (CIBEREHD), Instituto de Salud Carlos IIII, Madrid, Spain; ^4^Unidad de Investigación Hepática, Hospital Universitario Santa Cristina, Instituto de Investigación Sanitaria del Hospital Universitario de La Princesa, Madrid, Spain; ^5^Renal and Vascular Inflammation Lab, Health Research Institute-Fundacion Jimenez Diaz (IIS-FJD), Autonoma University of Madrid (UAM), Madrid, Spain; ^6^Centro de Investigación Biomédica en Red de Diabetes y Enfermedades Metabólicas Asociadas (CIBERDEM), Instituto de Salud Carlos III, Madrid, Spain; ^7^Department of Cell Biology, Physiology and Immunology, University of Cordoba, Cordoba, Spain; ^8^Maimonides Biomedical Research Institute of Cordoba (IMIBIC), University of Cordoba, Cordoba, Spain; ^9^Hospital Universitario Reina Sofia, Cordoba, Spain; ^10^Centre of Biomedical Research in Network of Cardiovascular Disease (CIBERCV), Madrid, Spain

**Keywords:** Nrf2, oxidative stress, inflammation, disease, injury

Oxidative stress and inflammation are pathological processes involved in the genesis and development of numerous disorders. Nuclear factor erythroid 2–related factor 2 (Nrf2) is a transcription factor that regulates the expression of multiple antioxidant and cytoprotective proteins and enzymes, thus playing a key role in protection against oxidative damage. There is a growing interest in understanding the specific role of Nrf2 in the development and progression of diseases as well as design novel approaches targeting Nrf2 to prevent and/or retard tissue injury.

This special issue contains 16 papers, including 7 original research articles and 9 reviews, reporting important data about the key role of Nrf2 in different pathological conditions.

In the article entitled “Nrf2 as a potential mediator of cardiovascular risk in metabolic diseases” by da Costa et al., a reduction in Nrf2 activity in diabetes, obesity, and atherosclerosis, thus increasing cardiovascular risk, was described. Importantly, systemic administration of specific Nrf2 inducers provided beneficial effects in cardiovascular diseases (da Costa et al.). In this sense, a wide range of natural products are promising therapeutic approaches to reduce atherogenesis progression by targeting the Nrf2 signaling pathway. These compounds activate Nrf2 by multiple mechanisms, such as targeting cysteine residues of Keap-1, disturbance of Nrf2/Keap1 interaction, epigenetic modification, and activation of protein kinases pathways. The beneficial effects of bioactive compounds are related to a reduction in reactive oxygen species (ROS) production, augmentation of glutathione levels, inhibition of nuclear factor kappa B inflammatory signaling pathway, and lowering foam cell production (Ooi et al.). Nrf2 activation endows antioxidant defense and reduces atherosclerosis risk in diabetes, where vascular lesions are a common complication of this disease. Tert-butyl hydroquinone (tBHQ) increased Nrf2 activation in vascular smooth cells and macrophages. Treatment with tBHQ decrease the size and extension of the atheroma plaque, besides lipid content, inflammation, foam cell size, and chemokine expression. An increase in the autophagic flux in the aorta and an upregulation of autophagy-related molecules after tBHQ treatment were also observed (Lazaro et al.). Another complication of chronic hyperglycemia is related to cognitive dysfunction. Nrf2 is involved in cellular homeostasis during diabetes mellitus. Astaxanthin, a potent antioxidant, exerted a protective effect on cognitive function through the inhibition of oxidative stress and inflammatory responses by the elevation of the Nrf2 antioxidant responsive element signaling pathway in the brains of chronic diabetic rats (Feng et al.). Nrf2 activation has been proposed as an antidiabetic strategy. In this context, naringenin, a bioactive flavonoid found in citrus fruits, protected pancreatic beta cells from oxidative damage *in vitro* and in streptozotocin-induced diabetic mice by activating Nrf2. Naringenin treatment decreased blood glucose levels, normalized lipid profile, restored insulin expression, promoted glycolysis, and inhibited gluconeogenesis in streptozotocin-induced diabetic mice (Rajappa et al.).

Oxidative stress and inflammation play a key role in the central nervous system physiology and pathophysiology of different diseases. The brain is highly susceptible to oxidative damage, so modulation of Nrf2 is important to provide beneficial effects in cerebrovascular diseases like ischemic stroke (Liu et al.). Free radical accumulation, due to impaired cellular antioxidant defenses or excessive production, results in neurotoxicity and cell death. Aging leads to a gradual increase in brain oxidative stress, which is accompanied by reduced antioxidant defenses, and a reduction in Nrf2 activity. Nrf2 could be modulated by dietary interventions (dietary energy restriction and high energy consumption) in animal models of neurological disorders, which leads to improving cognitive function, metabolic health, and longevity (Vasconcelos et al.). As oxidative stress is an important contributor of age-related macular degeneration, an increase in the antioxidant defenses can provide novel therapeutic strategies for this disease. Beneficial effects of Nrf2 activation have been described in retinal cells exposed to different types of oxidative stress inductors, so several natural and synthetic activators of Nrf2 have been tested as effective therapeutic agents for age-related macular degeneration (Bellezza).

Depression is a heterogeneous mood disorder characterized by mood alterations, social withdrawal, feelings of guilt, low self-esteem, anhedonia, idiopathic pain, loss of interest in enjoyable activities, and suicidal tendencies. Clinical and preclinical studies have revealed that oxidative stress and increased activity of immune factor cascades play significant roles in the pathophysiology of depression. Different studies published in this special issue have outlined the participation of Keap1-Nrf2 axis in depression (Hashimoto) and the role of quinolinic acid, an N-methyl-D-aspartate agonist, a pro-oxidant factor that reduce Nrf2 activity in depression (Bansal et al.).

Oxidative stress and inflammation are also a common feature of painful diseases. ROS are generated by enzymes of immune and nonimmune cells as part of protective actions. In this context, Staurengo-Ferrari et al. reviewed the involvement of Nrf2 in the mechanisms of action of classic analgesic and anti-inflammatory drugs, as well as natural products and other molecules that modulate Nrf2, in preclinical and clinical stages (Staurengo-Ferrari et al.). Chronic neuropathic pain is associated with anxiety- and depressive-like disorders. Interestingly, administration of sulforaphane (SFN), a Nrf2 inductor, had antinociceptive, anxiolytic, and antidepressant effects after the induction of persistent neuropathic pain (chronic constriction injury of the sciatic nerve) by normalizing oxidative stress and inhibiting microglial activation, which goes along with by the normalization of several biochemical parameters in the spinal cord. Moreover, SFN potentiated the antiallodynic effect of morphine (Ferreira-Chamorro et al.). In another article, administration of SFN 30 min before spared nerve injury surgery significantly reduced mechanical withdrawal threshold scores and sucrose preference, and restored tissue Keap1 and Nrf2 levels. This study suggests that decreased Keap1-Nrf2 signaling in the medial prefrontal cortex, hippocampus, and muscle may contribute to anhedonia susceptibility and that SFN exerts beneficial effects by normalization of decreased Keap1-Nrf2 signaling (Li et al.).

In another review submitted to this special issue, different therapeutic approaches for improving the prognosis of liver diseases, including acute liver failure, alcoholic and nonalcoholic fatty liver disease, viral hepatitis, and hepatocellular carcinoma by targeting hepatic Nrf2 are extensively described (Xu et al.). Besides liver diseases, Nrf2 activators can be used as potential therapies for other inflammatory disorders. For example, dinardokanshone C, a new compound isolated from the roots of *Nardostachys chinensis*, suppressed the induction of inflammatory markers such as cyclooxygenase 2, prostaglandin E2, tumor necrosis factor alpha, and interleukin 6 in lipopolysaccharide-stimulated macrophages, inhibiting M1 phenotype and ROS production, through the activation of Nrf2 pathway (Luo et al.).

Finally, the article by Rubio-Navarro et al. described that Nrf2 also plays an important role in renal protection against oxidative stress in renal diseases (Rubio-Navarro et al.). Massive intravascular hemolysis induces acute kidney injury. In this context, hemoglobin is accumulated in the kidney, inducing oxidative stress, inflammation, and tubular cell death. It has been demonstrated that Nrf2 plays an important role against hemoglobin-mediated renal damage. Thus, Nrf2 deficiency produces an increase in renal injury, loss of kidney function, oxidative and reticulum endoplasmic stress, and cell death. Moreover, Nrf2 activation by SFN treatment protected both *in vivo* and *in vitro* against hemoglobin toxicity.

In summary, this special issue will help to understand the important role of Nrf2 in disease. The articles published in this special issue show novel molecular mechanisms involved in Nrf2-mediated tissue protection as well as possible therapeutic approaches to decrease organ injury.

## Author Contributions

JE, AG-R, CG-G and JM wrote the manuscript. All authors critically revised the manuscript for important intellectual content.

## Funding

This is supported by FIS/FEDER CP14/00008, CP16/00014, CP16/00017, PI15/00448, PI16/00735, PI16/02057, PI17/00130, Spanish Ministry of Economy and Competitiveness (RYC-2017-22369), Sociedad Española de Nefrología, Fundacion Renal Iñigo Álvarez de Toledo (FRIAT). Centro de Investigación Biomédica en Red de Diabetes y Enfermedades Metabólicas Asociadas (CIBERDEM), Centro de Investigación Biomédica en Red Enfermedades Cardiovasculares (CIBERCV), Centro de Investigación Biomédica en Red de Enfermedades Hepáticas y Digestivas (CIBEREHD).

## Conflict of Interest

The authors declare that the research was conducted in the absence of any commercial or financial relationships that could be construed as a potential conflict of interest.

